# Agreement between three state-of-the-art deep learning bone age estimation models and chronological age in a large contemporary pediatric cohort

**DOI:** 10.1007/s00247-026-06661-8

**Published:** 2026-06-03

**Authors:** Elanchezhian Somasundaram, Rama S. Ayyala, William Tepe, Bryan Luna, Jonathan R. Dillman

**Affiliations:** Department of Radiology, Cincinnati Children’s Hospital Medical Center, 3333 Burnet Avenue, Cincinnati, OH 45229 United States

**Keywords:** Agreement, Artificial intelligence, Bias, Bone age, Children, Greulich and Pyle

## Abstract

**Background:**

Radiographic bone age estimation is routinely performed in children to evaluate short stature, early or late puberty, and endocrine disorders, as well as for surgical planning.

**Objective:**

To evaluate agreement between three state-of-the-art deep learning bone age estimation models and chronological age in a large contemporary pediatric cohort.

**Materials and methods:**

We retrospectively identified a large contemporary cohort of children (*n*=7,189; 3,669 females and 3,520 males) aged 24–216 months (mean 143.6±46.2 months) with consecutive radiologically normal hand (including the wrist) radiographs that were evaluated for trauma between November 1, 2010, and October 31, 2020. Bone age was estimated according to Greulich and Pyle (GP) Atlas standards using three state-of-the-art deep learning models (Stanford University, Cincinnati Children's Hospital Medical Center (CCHMC) , and MedImageInsight models), each producing continuous bone age estimates in months. Mean and proportional bias relative to chronological age were assessed.

**Results:**

All three models systematically overestimated chronological age, although the magnitude of bias varied by model and sex. The CCHMC model demonstrated the smallest overall mean bias (+3.20 months), followed by the MedImageInsight (+4.60 months) and Stanford (+7.03 months) models. Mean differences compared with chronological age were statistically significant for all models (*P*<0.0001). Evidence of proportional bias was observed in most models and subgroups. For all three models, the difference between predicted bone age and chronological age was greater for Black compared to White and Hispanic compared to non-Hispanic children.

**Conclusion:**

GP Atlas–based bone age models systematically overestimate chronological age in a large contemporary cohort of children undergoing hand radiography for trauma, with biases related to age, sex, race, and ethnicity.

**Supplementary Information:**

The online version contains supplementary material available at https://doi.org/10.1007/s00247-026-06661-8.

## Introduction

Radiographic bone age estimation is routinely performed in children to assess skeletal maturity. Indications include evaluation of short stature, early or late puberty, preoperative planning, and assessment and management of a range of endocrine disorders [1, 2]. Bone age is most commonly estimated using the radiographic hand and wrist Greulich and Pyle (GP) Atlas, first published in 1959 [3]. This atlas comprises 31 female and 29 male left hand (including the wrist) radiographs from infants, children, and adolescents. The reference standards were derived predominantly from White, upper-middle-class children in northern Ohio who participated in the Brush Foundation Growth Study between 1931 and 1942.

Increasing evidence suggests that physical child development is occurring earlier than in previous generations, including an earlier onset of puberty and menarche [4]. The underlying causes are not fully understood but likely include environmental influences, rising rates of obesity, and psychosocial stress [5]. Limited data are available comparing GP Atlas-based bone age estimates with actual chronological age in contemporary healthy children to determine if bias exists between estimated bone age and chronological age [6, 7].

This study aimed to assess agreement between three state-of-the-art deep learning models for bone age estimation and chronological age, as well as variability among models, in a large contemporary pediatric cohort undergoing hand radiography for trauma. The impacts of sex, race, and ethnicity on differences between bone age prediction and chronological age also were evaluated.

## Methods

This study was approved by the CCHMC institutional review board and was compliant with the Health Insurance Portability and Accountability Act. The requirement for informed consent was waived.

**Fig. 1:**
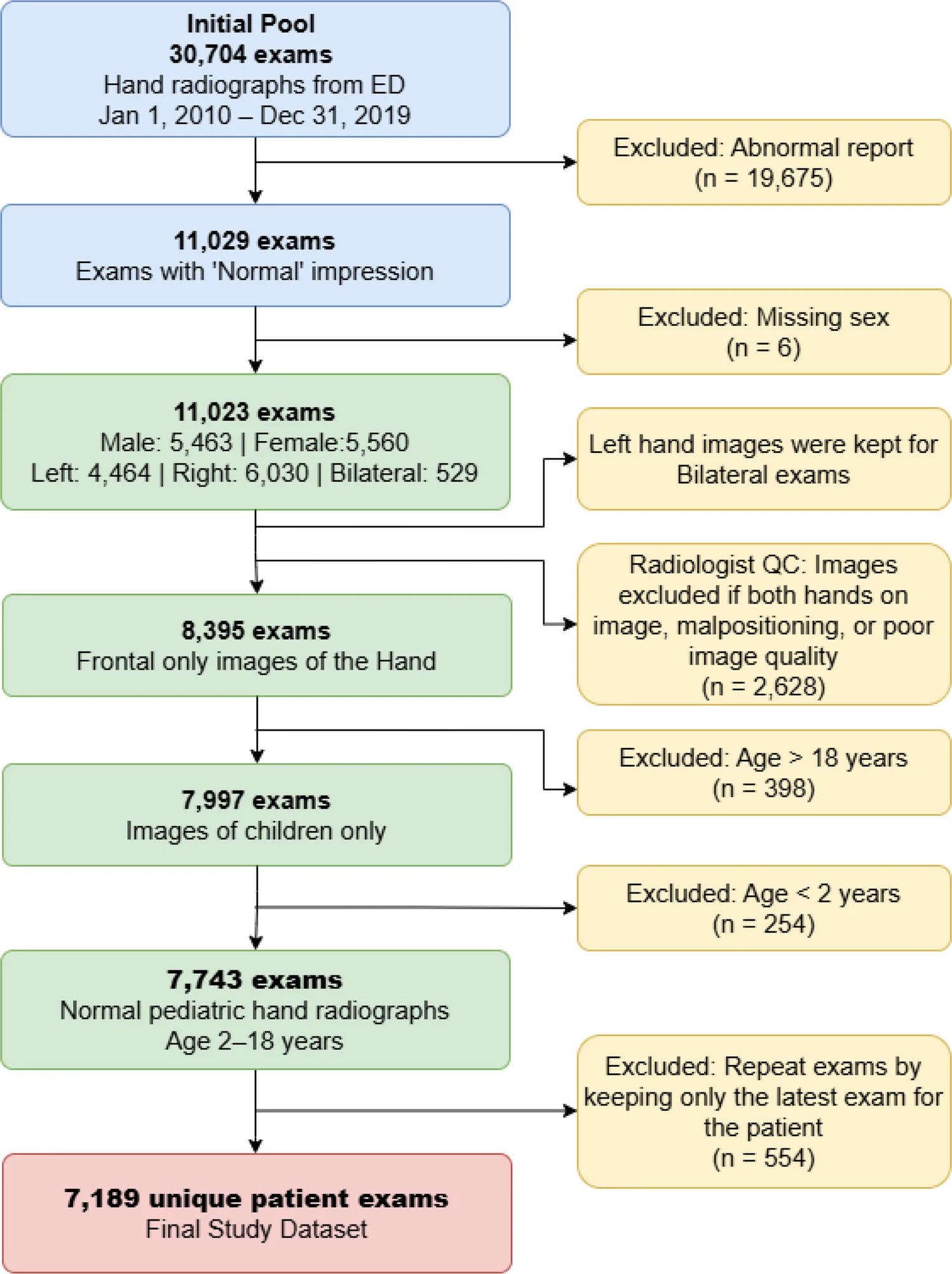
Participant flow diagram

We identified a large, contemporary cohort of children (*n*=7,189; 3,669 females and 3,520 males) aged 24–216 months (mean 143.6±46.2 months) who underwent consecutive right (*n*=4,103) or left (*n*=3,086) hand (including the wrist) radiography for evaluation of skeletal trauma between November 1, 2010, and October 31, 2020. Based on review of the “Impression” section of the clinical imaging reports, examinations with any reported radiologic abnormality were excluded. Only studies interpreted as entirely normal were included in our study. In patients with multiple radiographic examinations of the hand during the study period, only the most recent examination was used for our study, with any older examinations excluded. All potentially eligible imaging studies were manually reviewed by two board-certified, fellowship-trained pediatric radiologists to identify the frontal (as opposed to lateral or oblique) radiographs as well as to ensure sufficient anatomic coverage (distal forearm through fingertips) and overall diagnostic image quality. Frontal radiographs that were severely malpositioned, that contained both hands and wrists on the same image, or that were severely under- or overexposed were also excluded. A participant flow diagram is presented in Fig. 1.

Using electronic health records, patient sex, race, and ethnicity were documented. Chronological age at the time of hand radiography was also recorded for each patient.

For each participant, bone age was estimated according to the GP Atlas standards using three separate state-of-the-art deep learning models. These models were all trained on left hand radiographs from the RSNA Pediatric Bone Age Challenge dataset, which were annotated by radiologists using GP Atlas standards. Each model provides a continuous estimate of bone age and is described in more detail in the “Supplemental Methods” section: (1) Stanford University model (mean absolute error (MAE)=4.6 months, mean bias=0.04 months); (2) CCHMC model (MAE=4.7 months, mean bias=0.92 months); and (3) MedImageInsight model (MAE=6.2 months, mean bias=3.7 months). For each patient and model, the difference between estimated bone age and chronological age was calculated in months (i.e., the number of months by which the model over- or under-estimated chronological age). Estimated bone age and chronological age were rounded to the nearest month for our analyses. The MAE and mean bias for each model (in parentheses just above) were evaluated using the publicly available RSNA test dataset (*n*=200), which was labeled by expert radiologists according to the GP Atlas standards [8].

Continuous variables were summarized using means, standard deviations, and ranges. Categorical variables were summarized as counts and percentages. Bland–Altman plots were used to estimate agreement (i.e., mean bias) between model-derived bone age and chronological age for the overall cohort and stratified by sex, race (Black and White), and ethnicity (Hispanic and non-Hispanic); the biases for radiographs of the right and left hands were also separately evaluated. Linear regression of the Bland–Altman plots was performed to assess proportional bias (i.e., if any detected bias was consistent or changed with age). A two-sided *P*-value <0.05 was considered statistically significant. Statistical analyses were conducted using MedCalc Statistical Software version 20.111 (MedCalc Software Ltd, Ostend, Belgium; https://www.medcalc.org; 2022).

## Results

**Fig. 2:**
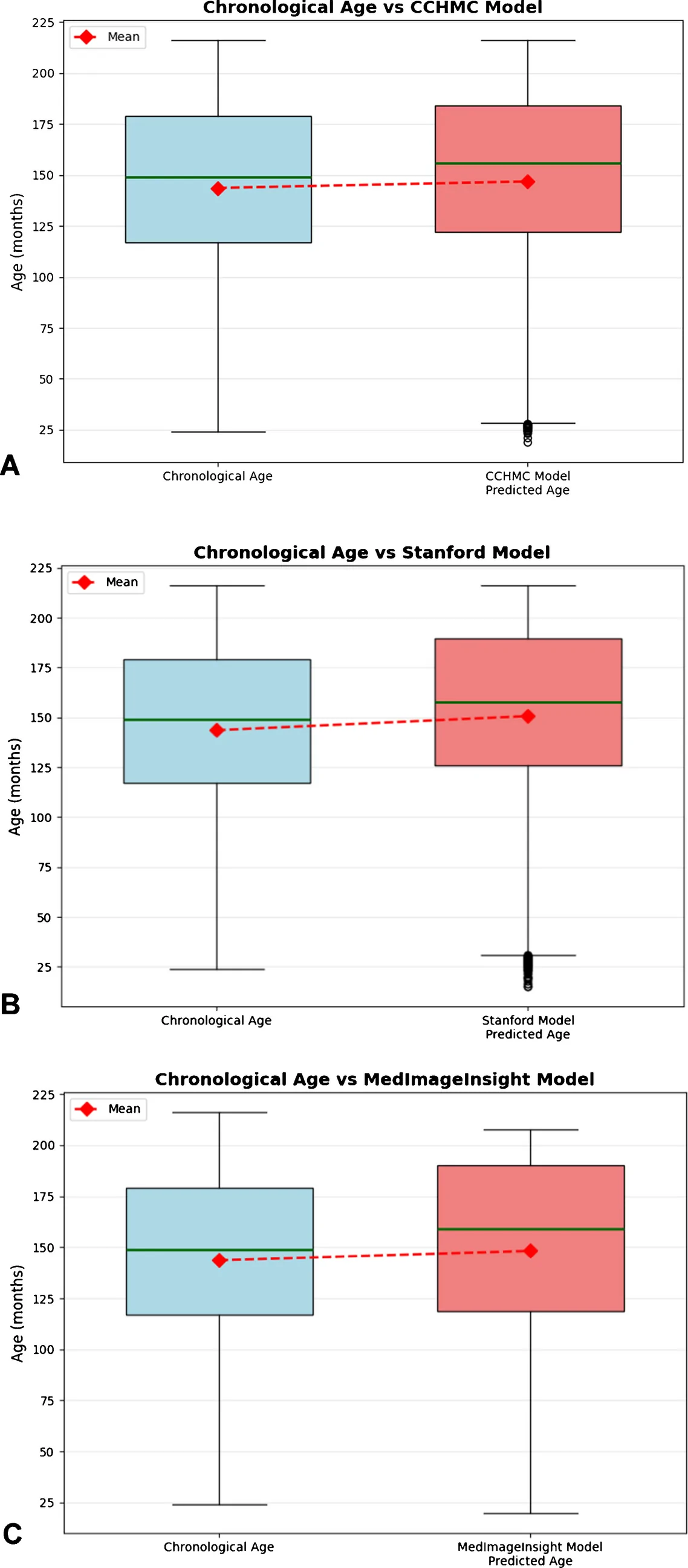
Tukey box plots showing distributions of chronological ages and deep learning model-predicted GP Atlas-based bone ages for the entire study sample (in months). **A** Chronological age and CCHMC model. **B** Chronological age and Stanford model. **C** Chronological age and MedImageInsight model. *Dotted red lines* connect the means of distributions

**Fig. 3:**
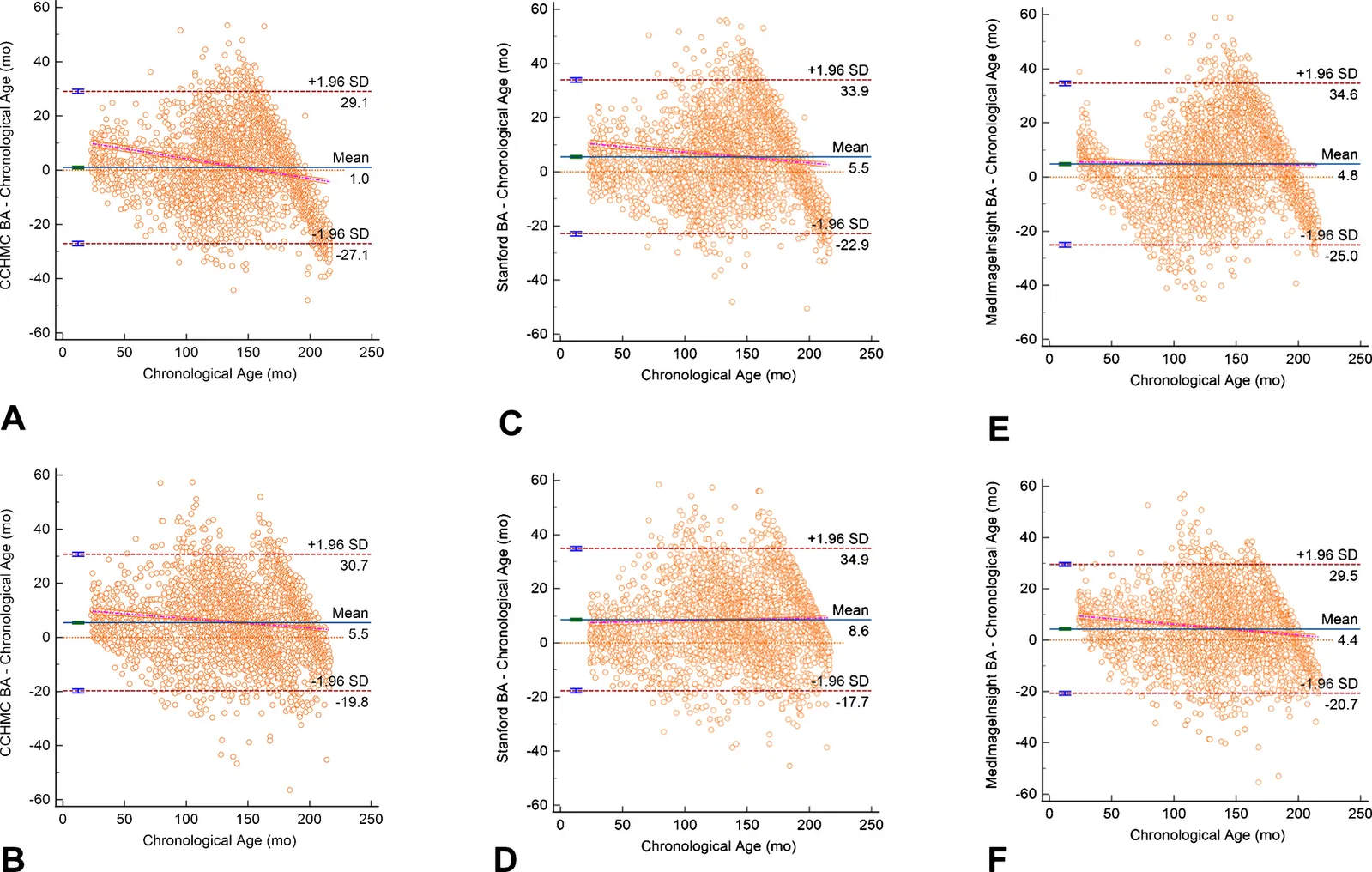
Bland-Altman plots comparing deep learning-predicted bone age (*BA*) benchmarked to Greulich and Pyle Atlas vs. chronological age (in months). **A**, **B** CCHMC model vs. chronological age for females and males. **C**, **D** Stanford model vs. chronological age for females and males. **E**, **F** MedImageInsight vs. chronological age for females and males. The *x-axis* is chronological age in months

Across the entire cohort, all three models systematically overestimated chronological age, although the magnitude of this mean bias varied by model and by sex (Table 1, Figs. 2 and 3). The CCHMC model demonstrated the smallest mean bias overall (+3.20 months), followed by MedImageInsight model (+4.60 months) and the Stanford model (+7.03 months). All three models showed statistically significant mean differences compared with chronological age for the entire cohort (*P*<0.0001 for all comparisons). The 95% limits of agreement were broad for all models, indicating substantial individual-level variability despite relatively small overall mean biases (Table 2).

**Table 1:** Study sample characteristics and bone age prediction model outputs (months)

Group	Chronologic age mean±SD (range) [IQR]	CCHMC mean±SD (range) [IQR]	Stanford mean±SD (range) [IQR]	MedImageInsight mean±SD (range) [IQR]
All (*n*=7,189)	143.6±46.2 (24–216) [117–179]	146.8±45.7 (18.9–216) [121.9–184.2]	150.7±47.6 (15.1–216) [126.1–189.6]	148.2±47.2 (19.8–207.8) [118.5–190.4]
Female (*n*=3,669)	144.1±45.3 (24–216) [118–179]	145.1±44.3 (18.9–216) [118.9–183.5]	149.7±45.8 (15.1–216) [124.6–189.3]	148.9±47.5 (19.8–205.4) [119.6–190.7]
Male (*n*=3,520)	143.1±47.0 (24-216) [117-180]	148.6±47.1 (25.7–216) [125.9–189.6]	151.7±49.3 (15.1–216) [128.3–193.1]	147.5±46.6 (27.8–207.8) [117.3–188.2]

**Table 2:** Bland–Altman analyses comparing deep learning model bone age predictions with chronological age for the overall study cohort and by sex

Model	Group	Mean bias, months (95% CI)	*P*-value	95% limits of agreement (months)	Proportional bias	Slope
**CCHMC**	All	3.20 (2.88 to 3.52)	<0.0001	−23.9 to 30.3	Yes (*P*<0.0002)	−0.05
	Female	1.00 (0.54 to 1.47)	<0.0001	−27.1 to 29.1	Yes (*P*<0.0001)	−0.07
	Male	5.48 (5.06 to 5.91)	<0.0001	−19.8 to 30.7	Yes (*P*<0.0001)	−0.04
**Stanford**	All	7.03 (6.70 to 7.35)	<0.0001	−20.5 to 34.6	Yes (*P*<0.0001)	−0.02
	Female	5.52 (5.05 to 5.99)	<0.0001	−22.9 to 33.9	Yes (*P*<0.0001)	−0.04
	Male	8.60 (8.16 to 9.04)	<0.0001	−17.7 to 34.9	No (*P*=0.053)	0.009
**MedImageInsight**	All	4.60 (4.27 to 4.93)	<0.0001	−23.0 to 32.2	Yes (*P*<0.0001)	−0.02
	Female	4.76 (4.27 to 5.25)	<0.0001	−25.0 to 34.6	No (*P*=0.23)	−0.01
	Male	4.43 (4.01 to 4.86)	<0.0001	−20.7 to 29.5	Yes (*P*<0.0001)	−0.04

For female patients, the CCHMC model showed the smallest mean bias (+1.00 months), while the Stanford (+5.52 months) and MedImageInsight (+4.76 months) models demonstrated larger overestimations, on average. For male patients, mean bias was greatest for the Stanford model (+8.60 months) and less for the CCHMC (+5.48 months) and MedImageInsight (+4.43 months) models (Table 2).

There was evidence of proportional bias being present for most models and subgroups. The CCHMC model showed proportional bias overall and in both female and male patients. The Stanford model demonstrated proportional bias overall and in female patients, but not in male patients. The MedImageInsight model showed proportional bias overall and in male patients, but not in female patients. Slope magnitudes and associated *P*-values are presented in Table 2.

### Impact of race, ethnicity, and sidedness

Bland–Altman analyses, including mean and proportional biases, based on race (Black and White), ethnicity (Hispanic and Non-Hispanic), and side of imaging are presented in Table 3. For all three models, the difference between predicted bone age and chronological age was greater for Black compared to White and Hispanic compared to non-Hispanic children. There was negligible (less than 1 month) difference between predicted bone age and chronological age when comparing left and right hand radiographs.

**Table 3:** Bland–Altman analyses comparing deep learning model bone age predictions with chronological age based on race, ethnicity, and side of imaging

Model	Group	Mean bias, months (95% CI)	*P*-value	95% limits of agreement (months)	Proportional bias	Slope
CCHMC	Black (*n*=1,594)	5.33 (4.62 to 6.04)	<0.0001	−24.2 to 33.6	Yes (*P*<0.0001)	−0.08
	White (*n*=5,182)	2.42 (2.06 to 2.79)	<0.0001	−24.1 to 29.0	Yes (*P*<0.0001)	−0.05
	Hispanic (*n*=216)	8.24 (6.27 to 10.20)	<0.0001	−20.5 to 37.0	No (*P*=0.09)	−0.03
	Non-Hispanic (*n*=6,933)	3.05 (2.72 to 3.37)	<0.0001	−23.9 to 30.0	Yes (*P*<0.0001)	−0.06
	Left (*n*=3,086)	3.61 (3.14 to 4.08)	<0.0001	−22.7 to 29.9	Yes (*P*<0.0001)	−0.03
	Right (*n*=4,103)	2.89 (2.45 to 3.32)	<0.0001	−24.8 to 30.6	Yes (*P*<0.0001)	−0.08
Stanford	Black (*n*=1,594)	9.33 (8.62 to 10.05)	<0.0001	−19.2 to 37.8	Yes (*P*<0.0001)	−0.04
	White (*n*=5,182)	6.22 (5.84 to 6.59)	<0.0001	−20.9 to 33.3	No (*P=*0.06)	−0.01
	Hispanic (*n*=216)	11.81 (9.79 to 13.82)	<0.0001	−17.7 to 41.3	No (*P=*0.90)	0.003
	Non-Hispanic (*n*=6,933)	6.89 (6.56 to 7.22)	<0.0001	−20.6 to 34.3	Yes (*P*<0.0001)	−0.02
	Left (*n*=3,086)	6.60 (6.10 to 7.11)	<0.0001	−21.5 to 34.7	No (*P=*0.85)	0.001
	Right (*n*=4,103)	7.35 (6.93 to 7.77)	<0.0001	−19.8 to 34.5	Yes (*P*<0.0001)	−0.03
MedImageInsight	Black (*n*=1,594)	6.20 (5.48 to 6.91)	<0.0001	−22.3 to 34.7	Yes (*P*<0.0001)	−0.06
	White (*n*=5,182)	3.97 (3.59 to 4.34)	<0.0001	−23.2 to 31.2	Yes (*P=*0.0008)	−0.01
	Hispanic (*n*=216)	10.17 (8.20 to 12.13)	<0.0001	−18.6 to 38.9	No (*P=*0.65)	−0.01
	Non-Hispanic (*n*=6,933)	4.43 (4.10 to 4.76)	<0.0001	−23.1 to 31.9	Yes (*P*<0.0001)	−0.02
	Left (*n*=3,086)	5.05 (4.55 to 5.55)	<0.0001	−22.7 to 32.8	Yes (*P=*0.01)	−0.01
	Right (*n*=4,103)	4.26 (3.82 to 4.69)	<0.0001	−23.2 to 31.8	Yes (*P*<0.0001)	−0.03

## Discussion

In this large contemporary pediatric cohort, deep learning-predicted bone age estimates generated using the GP Atlas framework systematically overestimated the patients’ chronological ages. Across all three deep learning models benchmarked to GP Atlas standards, children were predicted to be skeletally older than their true age, suggesting that contemporary children may demonstrate more advanced skeletal maturation relative to the historical reference population upon which the GP Atlas was developed [9]. These findings are also consistent with a large cohort study that found that among 71,341 female individuals the mean (SD) age at menarche decreased from 12.5 (1.6) years in 1950 to 1969 to 11.9 (1.5) years in 2000 to 2005 [4].

Overall mean bias ranged from +3.20 months to +7.03 months, with all differences highly statistically significant. Among the evaluated models, the CCHMC model demonstrated the smallest overall bias, overestimating chronological age by approximately 3 months on average. The MedImageInsight model overestimated age by nearly 5 months, while the Stanford model exhibited the greatest bias, overestimating by approximately 7 months. Although all three models showed a consistent directional trend toward overestimation, the variability in magnitude between models suggests that implementation details, training data composition, and calibration approaches meaningfully influence model performance, even when benchmarked to the same GP Atlas standard [10]. The relatively wide limits of agreement observed for each model may relate to other variables, including expected substantial individual-level variability. Different from our study, a previous study by Kim et al. found almost no mean bias between model-based bone age estimates and chronological age at two different institutions in South Korea (mean biases of −0.02 months and 0.14 months, respectively) [11].

Sex-specific analyses revealed additional important differences. For female patients, the CCHMC model demonstrated the smallest mean bias (+1.00 months), whereas the Stanford (+5.52 months) and MedImageInsight (+4.76 months) models showed larger overestimations. In male patients, mean bias was greatest for the Stanford model (+8.60 months) and lower for the CCHMC (+5.48 months) and MedImageInsight (+4.43 months) models. Notably, the CCHMC model demonstrated a pronounced sex disparity in bias (approximately 4.5 months), whereas the MedImageInsight model exhibited nearly balanced performance across sexes. These findings suggest that sex-specific growth patterns may not be equally captured across deep learning bone age prediction models [10]. Given the clinical implications of bone age assessment for growth disorders, endocrinologic evaluation, and treatment timing, even modest systematic biases—particularly those that differ by sex—could potentially have meaningful impact on patient management potentially moving treatment windows to earlier ages [1, 2, 9].

Interestingly, across all three models, the difference between predicted bone age and chronological age was greater in Black compared with White children and in Hispanic compared with non-Hispanic children. Although this finding may partly reflect the relatively smaller numbers of Black and Hispanic children in our cohort, the observed mean biases may be genuine and warrant further investigation to ensure optimal patient care and minimize the risk of inadvertent harm. In contrast, the difference between predicted bone age and chronological age was negligible (less than 1 month) when comparing right and left hand radiographs. This suggests that, for most individuals, either hand can probably be used for bone age estimation.

Importantly, we also observed evidence of proportional bias, indicating that the degree of overestimation may vary across the age spectrum rather than remaining constant. The magnitude of proportional bias varied by both the model used and patient sex. These patterns warrant further investigation, as proportional bias could reflect shifts in maturation tempo at specific developmental stages or limitations in the underlying GP Atlas framework when applied to modern populations [12]. Future work should explore age-stratified model optimization strategies and examine whether nonlinear correction models can mitigate these effects.

This study has several strengths. It includes a large, contemporary cohort of pediatric patients from a dedicated children’s hospital and evaluates bone age estimation using three independent, state-of-the-art deep learning models. The directional consistency of findings across models strengthens the conclusion that systematic overestimation is not individual model-specific but likely reflects discordance between current populations and the historical GP Atlas reference standard.

Our study also has limitations. The retrospective study design, while enabling a large sample size, may introduce selection bias (e.g., due to non-random sampling due to including only trauma patients in our study cohort). Additionally, the cohort represents patients from a single tertiary pediatric center, which may limit generalizability to other geographic or demographic populations. The three evaluated models are themselves imperfect, with mean absolute errors ranging from approximately 4 months to 6 months. However, in-house validation of these models on the publicly available RSNA bone age dataset demonstrated only slight mean bias (ranging from +0.04 months to +3.7 months), suggesting that the larger systematic biases observed here likely reflect population-level differences in chronological versus GP Atlas bone age standards rather than purely algorithmic error. While we only included radiographic examinations interpreted as normal in our study cohort, medical records were not reviewed to identify any patients with underlying medical conditions that might impact estimated bone age, such as endocrine disorders, hormonal therapies, or skeletal dysplasias. Similarly, neither Tanner stage, body mass index, nutritional status, nor socioeconomic status was considered as part of our study. Finally, we included trauma radiographs of both the left and right hands as part of our study cohort, while bone age estimation deep learning models were all trained on left hands.

## Conclusion

In conclusion, all evaluated models systematically overestimated chronological age in this contemporary cohort, with mean biases ranging from +3.20 months to +7.03 months. Sex-based differences were evident, with greater bias in boys for two of the three models. The difference between predicted bone age and chronological age was greater in Black compared with White children and in Hispanic compared with non-Hispanic children. The presence of proportional bias further suggests that discrepancies between skeletal and chronological age are not uniform across development. Further studies are needed to determine the causes of these multiple biases (e.g., if they are due to long-term secular trends versus population or model-related factors), the value of population-specific model training or optimization (including by sex, race, and ethnicity), the sources of variability between bone age estimation deep learning models, and if the currently employed GP Atlas-based framework remains optimal in present-day pediatric populations.

## Supplementary Information

Below is the link to the electronic supplementary material.

Supplementary Material 1 (DOCX 23.2 KB)

## Data Availability

No datasets were generated or analysed during the current study.
