# Supplementary material for: Agreement between three state-of-the-art deep learning bone age estimation models and chronological age in a large contemporary pediatric cohort
Source: Pediatr Radiol. 2026 Jun 3;56(10):2275–82. doi: 10.1007/s00247-026-06661-8 (PMC13578036; doi:10.1007/s00247-026-06661-8)
Supplement: Supplementary file 1 [file 247_2026_6661_MOESM1_ESM.docx]

**Supplemental Methods**

The **Cincinnati Children’s Hospital Medical Center** (**CCHMC) bone age prediction model** was trained using the MONAI framework on the RSNA Bone Age Challenge dataset [1] and employed an Xception [2] architecture with early fusion of patient sex. Patient sex was incorporated as an additional binary input channel (male = 1, female = 0) alongside the hand and wrist radiograph. A three-model ensemble was created by shuffling the original RSNA training and validation datasets during training. Preprocessing steps included image resizing with aspect-ratio–preserving padding and random augmentations addressing noise, orientation, and rotation.

The **Stanford University model [3]** was based on an Inception V3 architecture originally developed for the RSNA Pediatric Bone Age Challenge. This network uses late fusion for incorporating patient sex: the hand and wrist radiograph is processed through convolutional layers, while patient sex is input into a separate fully connected layer and later concatenated with extracted image features for final age prediction. Image preprocessing included contrast-limited adaptive histogram equalization (CLAHE) to enhance bone detail.

The **MedImageInsight model** [4] (Microsoft Health and Life Sciences) was pre-trained using a Unified Contrastive Learning objective with a DaViT architecture on a large corpus of 3.7 million medical images spanning 14 imaging domains, leveraging both the RSNA [1] and Greulich–Pyle (GP) [5] datasets for bone age training and framing bone age estimation as a retrieval-enabled regression task, respectively. Input hand radiographs were resized and intensity-normalized prior to inference; no task-specific fine-tuning was performed. During pre-training, patient sex and developmental age were encoded as standardized text templates, enabling the model to learn sex-specific maturation patterns. The model supports both image-to-image and image-to-text retrieval; in this study, an image-to-text retrieval strategy was used, with text prompts encoding patient sex and skeletal maturity. Six-month age bins spanning 1–18 years were used, and predicted probabilities across bins were combined to generate a weighted average bone age estimate.

For all models, an estimated bone age of 216 months (18 years-old) was used for any predictions greater than 216 months.

**Supplemental References**

1.	Halabi SS, Prevedello LM, Kalpathy-Cramer J, et al. The RSNA pediatric bone age machine learning challenge. Radiology 2019; 290:498-503

2.	Chollet F. Xception: Deep learning with depthwise separable convolutions. In: Proceedings of the IEEE conference on computer vision and pattern recognition, 2017:1251-1258

3.	Eng DK, Khandwala NB, Long J, et al. Artificial intelligence algorithm improves radiologist performance in skeletal age assessment: a prospective multicenter randomized controlled trial. Radiology 2021; 301:692-699

4.	Codella NC, Jin Y, Jain S, et al. Medimageinsight: An open-source embedding model for general domain medical imaging. arXiv preprint arXiv:241006542 2024;

5.	Greulich WW, Pyle SI. Radiographic atlas of skeletal development of the hand and wrist. American Journal of the Medical Sciences 1959; 238:393
